# Supplementary material for: Genetic and environmental influences on data missingness in developmental cognitive neuroscience
Source: Commun Psychol. 2026 Apr 22;4:70. doi: 10.1038/s44271-026-00457-0 (PMC13102918; doi:10.1038/s44271-026-00457-0)
Supplement: Supplementary file 2 — Supplementary Information [file 44271_2026_457_MOESM2_ESM.pdf]

Supplementary material

Genetic and environmental influences on data missingness in developmental cognitive neuroscience

Bussu G., Portugal A.M., Viktorsson C., Hardiansyah I., Falck-Ytter T.

Supplementary Methods 1.

For experiment-level missingness, we used a liability-threshold model, where the observed binary outcome reflects an underlying normally distributed liability L:

$$L = A + C + E$$

where A, C, and E are latent factors representing additive genetic, shared environmental, and unique environmental influences, respectively. The observed binary outcome reflects whether this latent liability exceeds a threshold.

For trial-level missingness, we fitted standard univariate ACE models:

$$Y = aA + cC + eE$$

with variance

$$Var(Y) = a^2 + c^2 + e^2$$

where a^2 = *additive genetic variance;* c^2 = *shared environmental variance*; and e^2 = *unique environmental variance*.

Nested models (AE, CE, E) were obtained by constraining the corresponding variance components to zero and compared using likelihood ratio tests.

Supplementary Table 1. Assumption testing for univariate model tested on the binary composite score for missing data, compared to the fully saturated model.

**Model**	**-2LL**	**# parameters**	***df***	**AIC**	**Δ LL**	**Δ df**	**p**
Fully saturated	774.34	6	588	786	-	-	-
Submodel 1	774.91	4	590	783	.57	2	.75
Submodel 2	778.60	3	591	785	4.26	3	.24

Abbreviations: -2LL = log-likelihood fit statistics; df = degrees of freedom; AIC = Akaike Information Criterion; **Δ** *LL = difference in log-likelihood fit statistics from the reference model;* **Δ** *df = difference in degrees of freedom from the reference model.*

Submodel 1: equality of means across twin order.

Submodel 2: equality of means and variances across twin order and zygosity.

Supplementary Table 2. Univariate ACE model fitting on the binary composite score for missing data, with sub-models.

**Model**	**-2LL**	**# parameters**	***df***	**AIC**	**Δ LL**	**Δ df**	**p**	**A**	**C**	**E**
ACE	778.60	4	592	787	-	-	-	.18<br>[0; .60]	22<br>[0; .52]	.59<br>[.40; .79]
AE	779.17	3	593	785	.57	1	.45	.44<br>[.23; .62]	-	.56<br>[.38; .77]
CE	778.89	3	593	785	.29	1	.59	-	.37<br>[.20; .53]	.63<br>[.47; .80]
E	795.66	2	594	800	17.1	2	$2.0*10^{-4}$	-	-	1 [1; 1]

Abbreviations: -2LL = log-likelihood fit statistics; df = degrees of freedom; AIC = Akaike Information Criterion; **Δ** *LL = difference in log-likelihood fit statistics from the reference model;* **Δ** *df = difference in degrees of freedom from the reference model; A = % variance explained by additive genetics; C = % variance explained by shared family environment; E = % variance explained by unique environment.*

AE model: additive genetic and unique environmental components (C fixed to zero).

CE model: shared and unique environmental components (A fixed to zero).

E model: unique environment only (A and C fixed to zero).

Supplementary Table 3. Assumption testing for univariate model tested on the EEG experiment-level missing data, compared to the fully saturated model.

**Model**	**-2LL**	**# parameters**	***df***	**AIC**	**Δ LL**	**Δ df**	**p**
Fully saturated	642.99	6	588	655	-	-	-
Submodel 1	643.63	4	590	652	.63	2	.73
Submodel 2	646.47	3	591	652	3.44	3	.33

Abbreviations: -2LL = log-likelihood fit statistics; df = degrees of freedom; AIC = Akaike Information Criterion; **Δ** *LL = difference in log-likelihood fit statistics from the reference model;* **Δ** *df = difference in degrees of freedom from the reference model.*

Submodel 1: equality of means across twin order.

Submodel 2: equality of means and variances across twin order and zygosity.

Supplementary Table 4. Assumption testing for univariate model tested on the gaze-tracking experiment-level missing data, compared to the fully saturated model.

**Model**	**-2LL**	**# parameters**	***df***	**AIC**	**Δ LL**	**Δ df**	**p**
Fully saturated	369.37	6	588	381	-	-	-
Submodel 1	372.68	4	590	381	3.31	2	.19
Submodel 2	372.73	3	591	378	3.36	3	.34

Abbreviations: -2LL = log-likelihood fit statistics; df = degrees of freedom; AIC = Akaike Information Criterion; **Δ** *LL = difference in log-likelihood fit statistics from the reference model;* **Δ** *df = difference in degrees of freedom from the reference model.*

Submodel 1: equality of means across twin order.

Submodel 2: equality of means and variances across twin order and zygosity.

Supplementary Table 5. Assumption testing for univariate model tested on the pupillometry experiment-level missing data, compared to the fully saturated model.

**Model**	**-2LL**	**# parameters**	***df***	**AIC**	**Δ LL**	**Δ df**	**p**
Fully saturated	454.07	6	588	466	-	-	-
Submodel 1	454.17	4	590	462	.10	2	.95
Submodel 2	454.40	3	591	460	.33	3	.95

Abbreviations: -2LL = log-likelihood fit statistics; df = degrees of freedom; AIC = Akaike Information Criterion; **Δ** *LL = difference in log-likelihood fit statistics from the reference model;* **Δ** *df = difference in degrees of freedom from the reference model.*

Submodel 1: equality of means across twin order.

Submodel 2: equality of means and variances across twin order and zygosity.

Supplementary Table 6. Univariate ACE model fitting on the EEG experiment-level missing data, with sub-models.

**Model**	**-2LL**	**# parameters**	***df***	**AIC**	**Δ LL**	**Δ df**	**p**	**A**	**C**	**E**
ACE	646.44	4	592	654	-	-	-	.42	.03	.56
								[0; .64]	[0; .52]	[.36; .80]
AE	646.44	3	593	652	.005	1	.94	.45	-	.55
								[.22; .64]		[.36; .78]
CE	647.51	3	593	654	1.07	1	.30	-	.37	.63
									[.17; .54]	[.46; .83]
E	660.25	2	594	664	13.8	2	.001	-	-	1 [1; 1]

Abbreviations: -2LL = log-likelihood fit statistics; df = degrees of freedom; AIC = Akaike Information Criterion; **Δ** *LL = difference in log-likelihood fit statistics from the reference model;* **Δ** *df = difference in degrees of freedom from the reference model; A = % variance explained by additive genetics; C = % variance explained by shared family environment; E = % variance explained by unique environment.*

AE model: additive genetic and unique environmental components (C fixed to zero).

CE model: shared and unique environmental components (A fixed to zero).

E model: unique environment only (A and C fixed to zero).

Supplementary Table 7. Univariate ACE model fitting on the gaze-tracking experiment-level missing data, with sub-models.

**Model**	**-2LL**	**# parameters**	***df***	**AIC**	**Δ LL**	**Δ df**	**p**	**A**	**C**	**E**
ACE	372.73	4	592	381	-	-	-	.03<br>[0; .75]	.46<br>[0; .70]	.51<br>[.24; .79]
AE	373.87	3	593	380	1.13	1	.29	.55<br>[.23; .78]	-	.45<br>[.22; .77]
CE	372.74	3	593	379	.003	1	.96	-	.48<br>[.21; .70]	.52<br>[.30; .79]
E	384.44	2	594	388	11.7	2	.003	-	-	1 [1; 1]

Abbreviations: -2LL = log-likelihood fit statistics; df = degrees of freedom; AIC = Akaike Information Criterion; ***Δ*** *LL = difference in log-likelihood fit statistics from the reference model;* ***Δ*** *df = difference in degrees of freedom from the reference model; A = % variance explained by additive genetics; C = % variance explained by shared family environment; E = % variance explained by unique environment.*

AE model: additive genetic and unique environmental components (C fixed to zero).

CE model: shared and unique environmental components (A fixed to zero).

E model: unique environment only (A and C fixed to zero).

Supplementary Table 8. Univariate ACE model fitting on the pupillometry experiment-level missing data, with sub-models.

**Model**	**-2LL**	**# parameters**	***df***	**AIC**	**Δ LL**	**Δ df**	**p**	**A**	**C**	**E**
ACE	454.40	4	592	462	-	-	-	.19<br>[0; .81]	.46<br>[0; .76]	.34<br>[.17; .57]
AE	456.14	3	593	462	1.75	1	.19	.69<br>[.47; .85]	-	.31<br>[.15; .53]
CE	454.67	3	593	461	.27	1	.60	-	.62<br>[.42; .77]	.38<br>[.23; .58]
E	484.14	2	594	488	29.7	2	$3.5*10^{-7}$	-	-	1 [1; 1]

Abbreviations: -2LL = log-likelihood fit statistics; df = degrees of freedom; AIC = Akaike Information Criterion; **Δ** *LL = difference in log-likelihood fit statistics from the reference model;* **Δ** *df = difference in degrees of freedom from the reference model; A = % variance explained by additive genetics; C = % variance explained by shared family environment; E = % variance explained by unique environment.*

AE model: additive genetic and unique environmental components (C fixed to zero).

CE model: shared and unique environmental components (A fixed to zero).

E model: unique environment only (A and C fixed to zero).

Supplementary Table 9. Assumption testing for univariate model tested on the number of valid trials provided for the gaze tracking experiment, compared to the fully saturated model.

**Model**	**-2LL**	**# parameters**	***df***	**AIC**	**Δ LL**	**Δ df**	**p**
Fully saturated	1608.01	10	565	1628.01	-	-	-
Submodel 1	1609.36	8	567	1625.36	1.34	2	.51
Submodel 2	1613.84	7	568	1627.84	5.83	3	.12
Submodel 3	1613.91	5	570	1623.91	5.89	5	.32
Submodel 4	1614.77	4	571	1622.77	6.76	6	.34

Abbreviations: -2LL = log-likelihood fit statistics; df = degrees of freedom; BIC = Bayesian Information Criterion; **Δ** *LL = difference in log-likelihood fit statistics from the reference model;* **Δ** *df = difference in degrees of freedom from the reference model.*

Submodel 1: equality of means across twin order.

Submodel 2: equality of means across twin order and zygosity.

Submodel 3: equality of means and variances across twin order.

Submodel 4: equality of means and variances across twin order and zygosity.

Supplementary Table 10. Assumption testing for univariate model tested on the number of trials provided for the EEG experiment, compared to the fully saturated model.

Model	-2LL	# parameters	*df*	AIC	Δ LL	Δ df	p
Fully saturated	1585.59	10	558	1605.59	-	-	-
Submodel 1	1587.09	8	560	1603.09	1.50	2	.47
Submodel 2	1587.68	7	561	1601.68	2.09	3	.55
Submodel 3	1588.40	5	563	1598.40	2.81	5	.73
Submodel 4	1588.40	4	564	1596.40	2.81	6	.83

Abbreviations: -2LL = log-likelihood fit statistics; df = degrees of freedom; BIC = Bayesian Information Criterion; **Δ** *LL = difference in log-likelihood fit statistics from the reference model;* **Δ** *df = difference in degrees of freedom from the reference model.*

Submodel 1: equality of means across twin order.

Submodel 2: equality of means across twin order and zygosity.

Submodel 3: equality of means and variances across twin order.

Submodel 4: equality of means and variances across twin order and zygosity.

Supplementary Table 11. Assumption testing for univariate model tested on the number of valid trials provided for the pupillometry experiment, compared to the fully saturated model.

**Model**	**-2LL**	**# parameters**	***df***	**AIC**	**Δ LL**	**Δ df**	**p**
Fully saturated	1560.84	10	561	1580.84	-	-	-
Submodel 1	1561.14	8	563	1577.14	0.30	2	.86
Submodel 2	1561.62	7	564	1575.62	0.78	3	.85
Submodel 3	1562.18	5	566	1572.18	1.34	5	.93
Submodel 4	1563.01	4	567	1571.01	2.17	6	.90

Abbreviations: -2LL = log-likelihood fit statistics; df = degrees of freedom; BIC = Bayesian Information Criterion; **Δ** *LL = difference in log-likelihood fit statistics from the reference model;* **Δ** *df = difference in degrees of freedom from the reference model.*

Submodel 1: equality of means across twin order.

Submodel 2: equality of means across twin order and zygosity.

Submodel 3: equality of means and variances across twin order.

Submodel 4: equality of means and variances across twin order and zygosity.

Supplementary Table 12. Descriptive measures for questionnaire and autism-related data, presented as *mean (standard deviation*).

		DZ (n=264)	MZ (n=330)
**Concurrent measures** (5 months of age)			
**VABS Social-Communication**	Mean (SD)	34.3 (1.8)	34.2 (1.5)
**VABS Motor**	Mean (SD)	10.4 (1.1)	10.2 (1.0)
**IBQ Surgency**	Mean (SD)	3.9 (0.8)	3.9 (0.8)
**IBQ Negative Affect**	Mean (SD)	2.9 (0.8)	3.0 (0.8)
**IBQ Effortful Control**	Mean (SD)	5.2 (0.7)	5.2 (0.6)
**ITSP Low Registration**	Mean (SD)	27.0 (5.8)	27.3 (5.3)
**ITSP Sensation Seeking**	Mean (SD)	25.5 (3.7)	24.8 (4.0)
**ITSP Sensory Sensitivity**	Mean (SD)	22.6 (6.3)	22.5 (5.6)
**ITSP Sensation Avoiding**	Mean (SD)	8.4 (3.0)	8.8 (2.9)
**Autism-related measures**			
**Polygenic Score for Autism**	Mean (SD)	-0.1 (1.1)	0.1 (0.9)
**Q-CHAT Total Score** (36 months of age)	Mean (SD)	21.3 (7.6)	22.3 (7.7)

Abbreviations: DZ = dizygotic twins; MZ = monozygotic twins; VABS = Vineland Adaptive Behavior Scales; IBQ = Infant Behavior Questionnaire (short form); ITSP = Infant/Toddler Sensory Profile; QCHAT = Quantitative Checklist for Autism Traits.

Supplementary Table 13. Associations between missingness and other traits

Demographic measures	Gaze tracking	EEG	Pupillometry
**Age** (in months)	.06 (.04); .14	.05 (.05); .33	.12 (.06); .04
**Sex** (Reference level: female)	.02 (.09); .80	.06 (.10); .54	-.04 (.10); .72
**Mean parental age** (in years)	-.08 (.04); .06	-.07 (.05); .10	$-4.9*10^{-3}$ (.05); .92
**Mean parental education**	-.08 (.04); .06	$-3.8*10^{-3}$ (.05); .94	-.07 (.05); .20
**Family income**	-.06 (.04); .18	$8.5*10^{-3}$ (.05); .85	-.05 (.05); .32
**Gestation age** (in months)	.11 (.05); .03	.04 (.04); .37	.14 (.05); $4.7*10^{-3}$
**Concurrent measures** (5 months of age)			
**VABS Social-Communication**	.07 (.04); .10	-.03 (.05); .54	.14 (.05); $3.56*10^{-3}$
**VABS Motor**	.09 (.05); .05	-.08 (.05); .10	.13 (.05); $9.15*10^{-3}$
**IBQ Surgency**	.09 (.05); .05	-.05 (.05); .30	.11 (.05); .015
**IBQ Negative Affect**	$-8.7*10^{-3}$ (.04); .85	-.05 (.05); .29	-.04 (.05); .41
**IBQ Effortful Control**	.06 (.04); .12	.04 (.04); .42	.10 (.05); .03
**ITSP Sensory Sensitivity**	-.05 (.05); .29	.05 (.05); .27	-.03 (.05); .63
**ITSP Sensation Avoiding**	.02 (.04); .71	-.02 (.05); .74	-.02 (.05); .73
**ITSP Sensation Seeking**	.02 (.05); .64	.05 (.05); .30	.02 (.05); .70
**ITSP Low Registration**	$-8.1*10^{-4}$ (.04); .98	$-4.3*10^{-3}$ (.04); .92	-.02 (.05); .56
**Autism-related measures**			
**Polygenic Score for Autism**	-.03 (.05); .58	.03 (.05); .46	.06 (.05); .28
**QCHAT Total Score** (36 months of age)	$2.5*10^{-3}$ (.05); .96	-.01 (.05); .78	.03 (.04); .56

Results from independent regression models through generalized estimated equations are listed for each quantitative score indexing data quality (i.e., trial-level missingness) across the three experiments (independent variable) and each phenotypic measure tested (independent variable). Results are shown as *regression coefficient (standard error); p-value.*

Of note, none of the associations tested survived Bonferroni correction for multiple comparisons (adjusted *alpha-level=0.0029* for each experimental hypothesis tested).

Abbreviations: EEG = electroencephalography); VABS = Vineland Adaptive Behavior Scales; IBQ = Infant Behavior Questionnaire (short form); ITSP = Infant/Toddler Sensory Profile; QCHAT = Quantitative Checklist for Autism Traits.
